# Supplementary material for: Selenomonas sputigena acts as a pathobiont mediating spatial structure and biofilm virulence in early childhood caries
Source: Nat Commun. 2023 May 22;14:2919. doi: 10.1038/s41467-023-38346-3 (PMC10202936; doi:10.1038/s41467-023-38346-3)
Supplement: Supplementary file 1 — Supplementary Information [file 41467_2023_38346_MOESM1_ESM.pdf]

Supplementary Figures and Tables

***Selenomonas sputigena* acts as a pathobiont mediating spatial structure and biofilm virulence in early childhood caries.**

Hunyong Cho*, Zhi Ren*, Kimon Divaris†, Jeffrey Roach, Bridget M. Lin, Chuwen Liu, M. Andrea Azcarate-Peril, Miguel A. Simancas-Pallares, Poojan Shrestha, Alena Orlenko, Jeannie Ginnis, Kari E. North, Andrea G. Ferreira Zandona, Apoena Aguiar Ribeiro, Di Wu† and Hyun Koo†

Supplementary Fig. 1 | Estimates of association between dental caries experience and the abundance of 16 significant species in the discovery (n = 300 children) and the replication samples (n = 116 children), illustrating the correspondence between coefficients in MTG and MTX analyses. The magnitude of association was measured by log-normal model coefficients (the increment of the log-abundance or log-expression level of each species per increment in the quantitative caries status) and a two-sided Wald test. Nominal p-values are presented in parentheses, with *, **, and *** denoting nominal *p*-values less than 0.1, 0.05, 0.01, respectively, and each pair of symbols next to the species names is a combination of metagenomics and metatranscriptomics results separated by /. The effect sizes and their 95% confidence intervals are available in the source data file in Supplementary Information. The diagonal lines represent $y = x$. The 4 underlined taxa are the "top species" that were prioritized for *in vitro* virulence assays, biofilm studies, and potential *in vivo* experiments. Each color represents a genus.

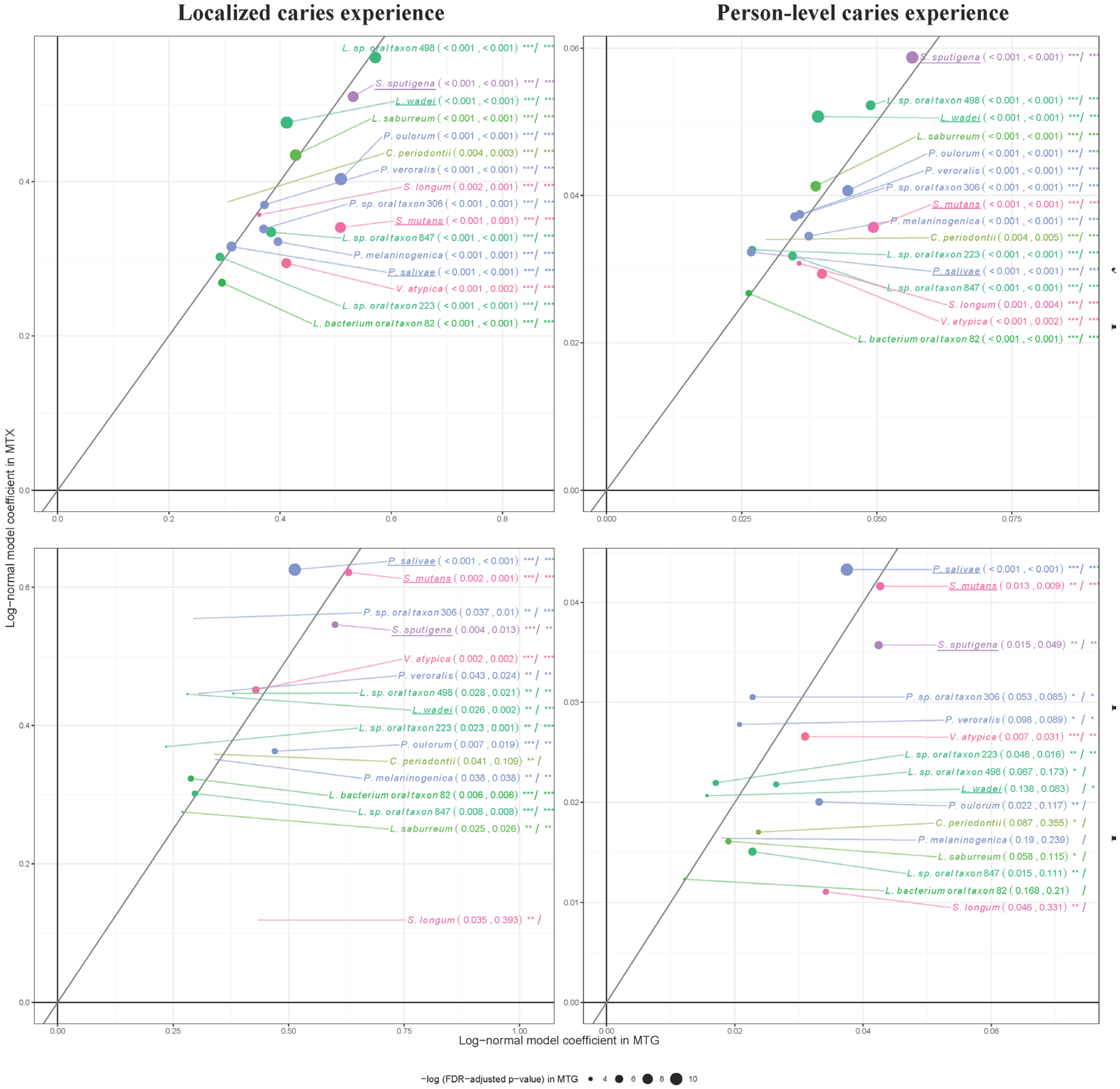

Supplementary Fig. 2 | Pathways involving the 16 significant species (top 30 in terms of proportional involvement among the top 100 most abundant pathways) identified in taxonomic analyses (i.e., % significant species on the *x*-axis) and their association with caries experience (*y*-axis). The magnitude of association was measured by log-normal model coefficients (the increment of the log-expression level of each pathway per the increment in the quantitative caries status), and statistical significance was determined with two-sided Wald tests and its FDR-adjusted *p*-values. Four pathways' relative expression in MTX was significantly associated with caries experience: glycolysis IV (ranked #3 overall in terms of expression in MTX data), pyruvate fermentation to acetate and lactate II (ranked #9), chorismate biosynthesis from 3-dehydroquinate and UDP-N-acetyl-D-glucosamine biosynthesis I. Lactose and galactose degradation I, although not significantly associated with caries experience after FDR correction, was the pathway with the highest proportional participation of significant species (38%, *L. wadei* and *S. mutans*).

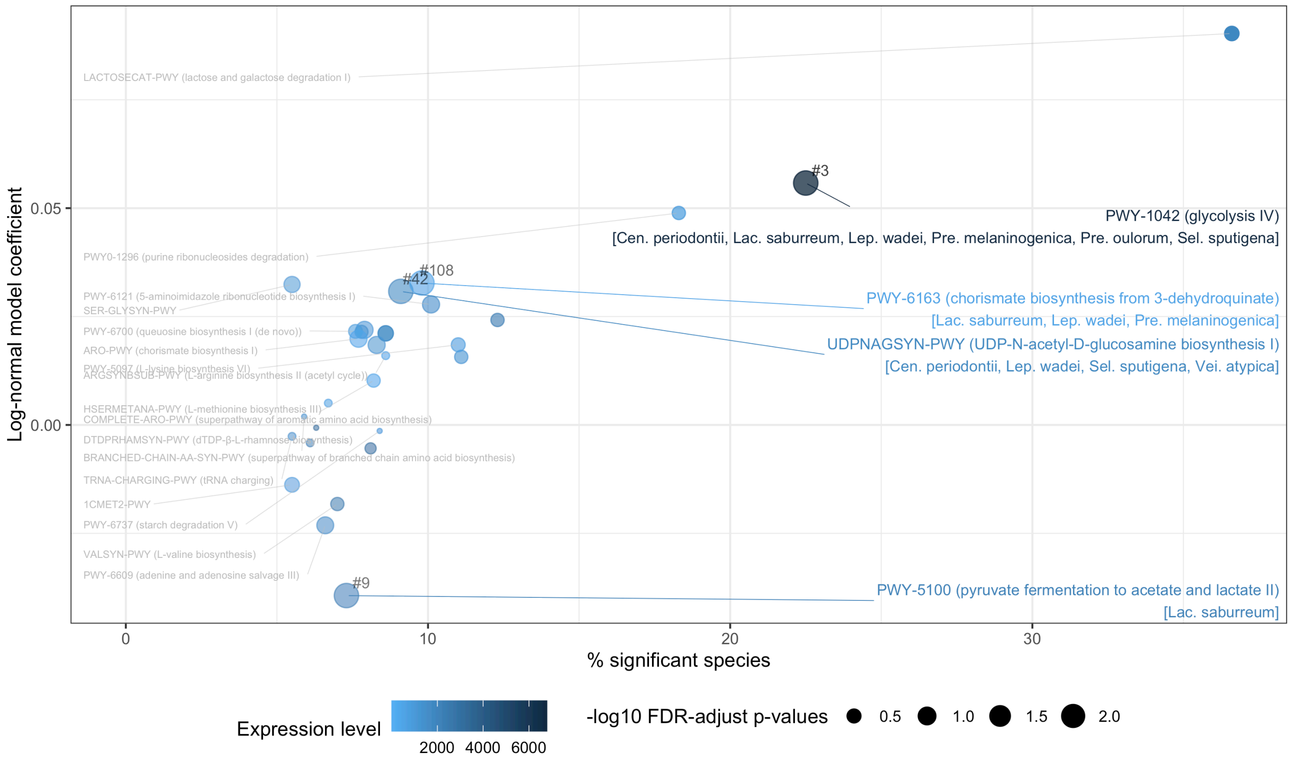

Supplementary Fig. 3 | A time course of biofilm development on tooth-mimetic surface. Confocal images (top views) of single- and mixed-species biofilms on saliva-coated hydroxyapatite surfaces formed by each of the new species alone and when co-cultured with *S. mutans*. Upper panel: Initial attachment stage at 4 hours. The hydroxyapatite surfaces are shown in gray. Middle panel: Intermediate stage at 10 hours. White arrowheads, *S. sputigena* cells forming aggregates in close proximity to *S. mutans* clusters. Lower panel: The mature biofilms at 24 hours (as shown in Fig. 5). In each panel, the upper images are single-species biofilms formed by *S. mutans* and each of the new top species, and the lower images are mixed biofilms of each new species co-cultured with *S. mutans*. Green, *S. mutans*; red, new candidate species (*S. sputigena, L. wadei* or *P. salivae*). Representative images from three independent experiments are shown. Scale bars, 20 μm.

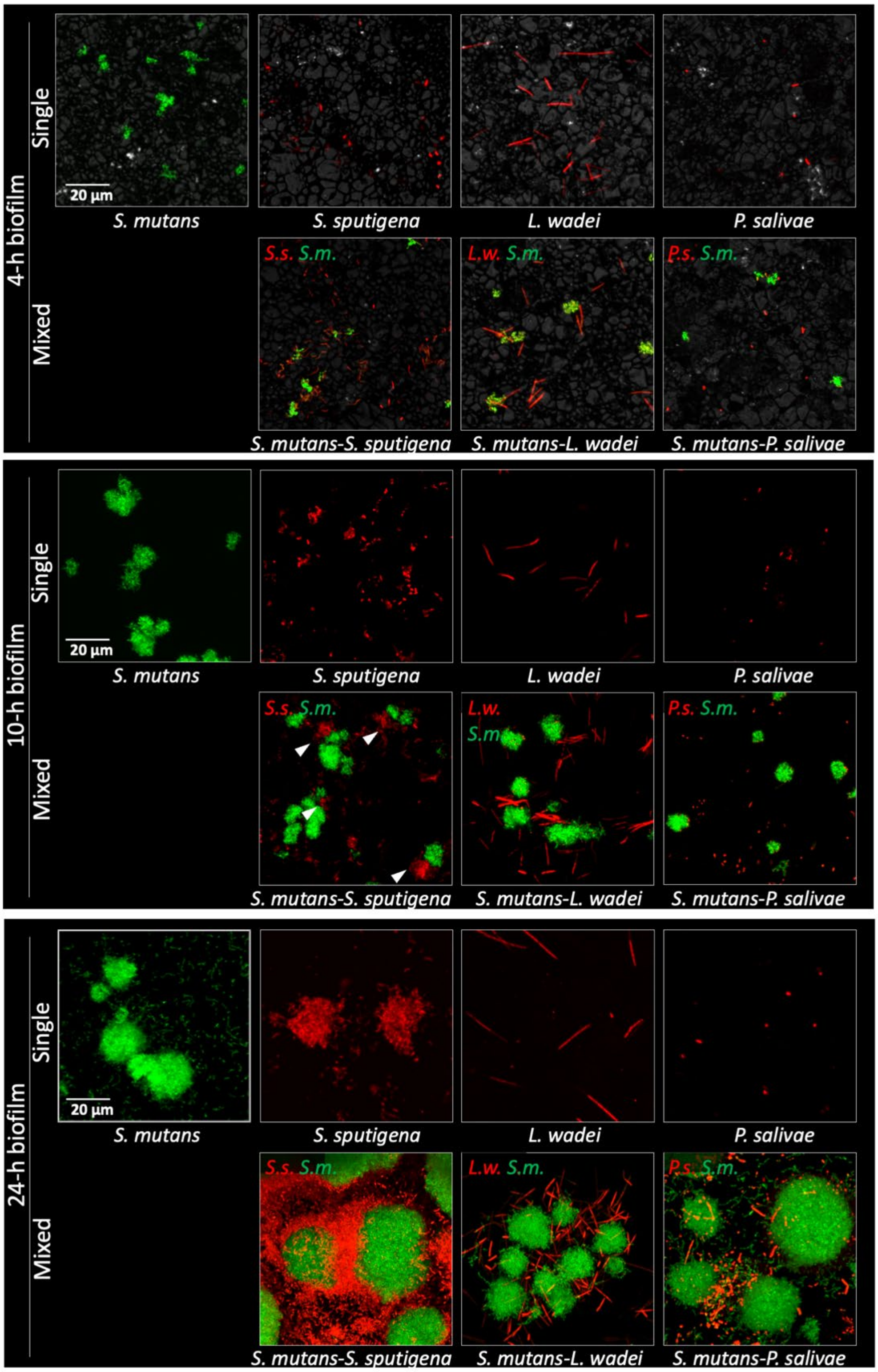

Supplementary Fig. 4 | *S. sputigena* in the single-species biofilm becomes trapped in extracellular glucans produced by exogenous, cell-free GtfB exoenzymes. (A) Immobilized *S. sputigena* cells trapped by EPS α-glucan matrix produced by purified GtfB. Red, *S. sputigena*; cyan, α-glucan matrix. Colors indicate trajectories that originated from individual cells. Top panel, *S. sputigena* cells trapped by GtfB produced α-glucan matrix showed no mobility; bottom panel, without GtfB, *S. sputigena* cells displayed surface mobility. (B) Accumulated *S. sputigena* cell displacement (total path length) relative to the initial position. Left, accumulated displacement of *S. sputigena* cells in the presence of GtfB; right, accumulated cell displacement in the vehicle control. SS, *S. sputigena*. Representative images from three independent experiments are shown. Scale bar, 10 µm.

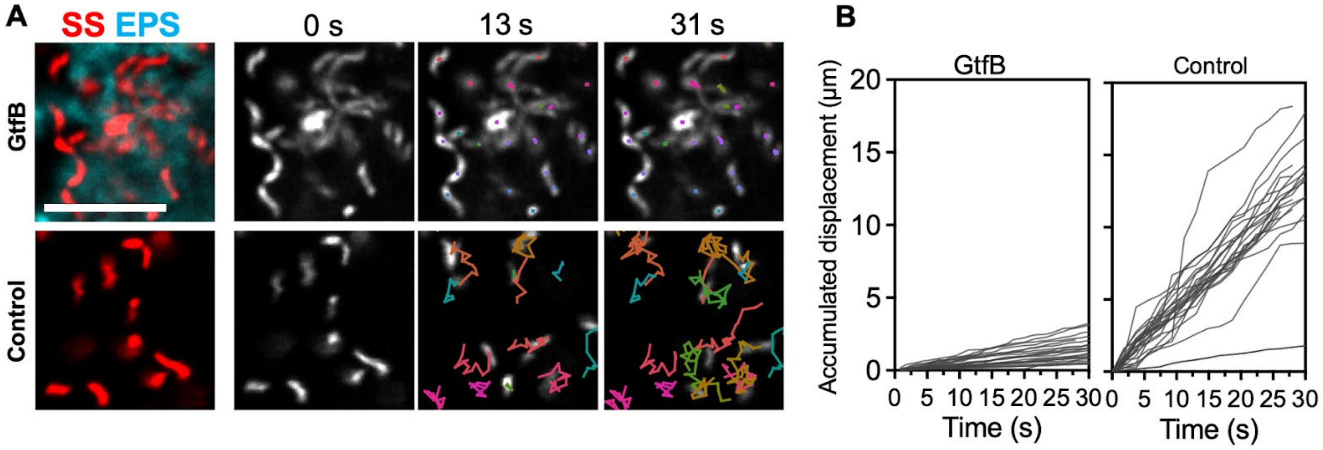

Supplementary Fig. 5 | Real-time pH profile at the biofilm-apatite interface. (A) *In situ* pH was assessed using a pH-responsive probe (C-SNARF-4) and high-resolution confocal imaging. The left image illustrates the biofilm structure on the surface. Images on the right show the real-time pH distribution at the interface (color-coded, pH 4.5-7.4) over time after 1% glucose exposure. White solid lines indicate the outline of the biofilm superstructure formed by *S. sputigena* and *S. mutans*. SM, *S. mutans*; SS, *S. sputigena.* Scale bar, 10 µm. (B) Quantitative pH measurements at the interface over time by ratiometric analysis. Data are plotted as mean ± standard deviation (n=3 biologically independent samples) and significant *p*-values are provided above the bars ($p<0.05$ by one-way analysis of variance with Tukey's multiple-comparison test at t=15 min). ns denotes differences not statistically significant ($p>0.05$).

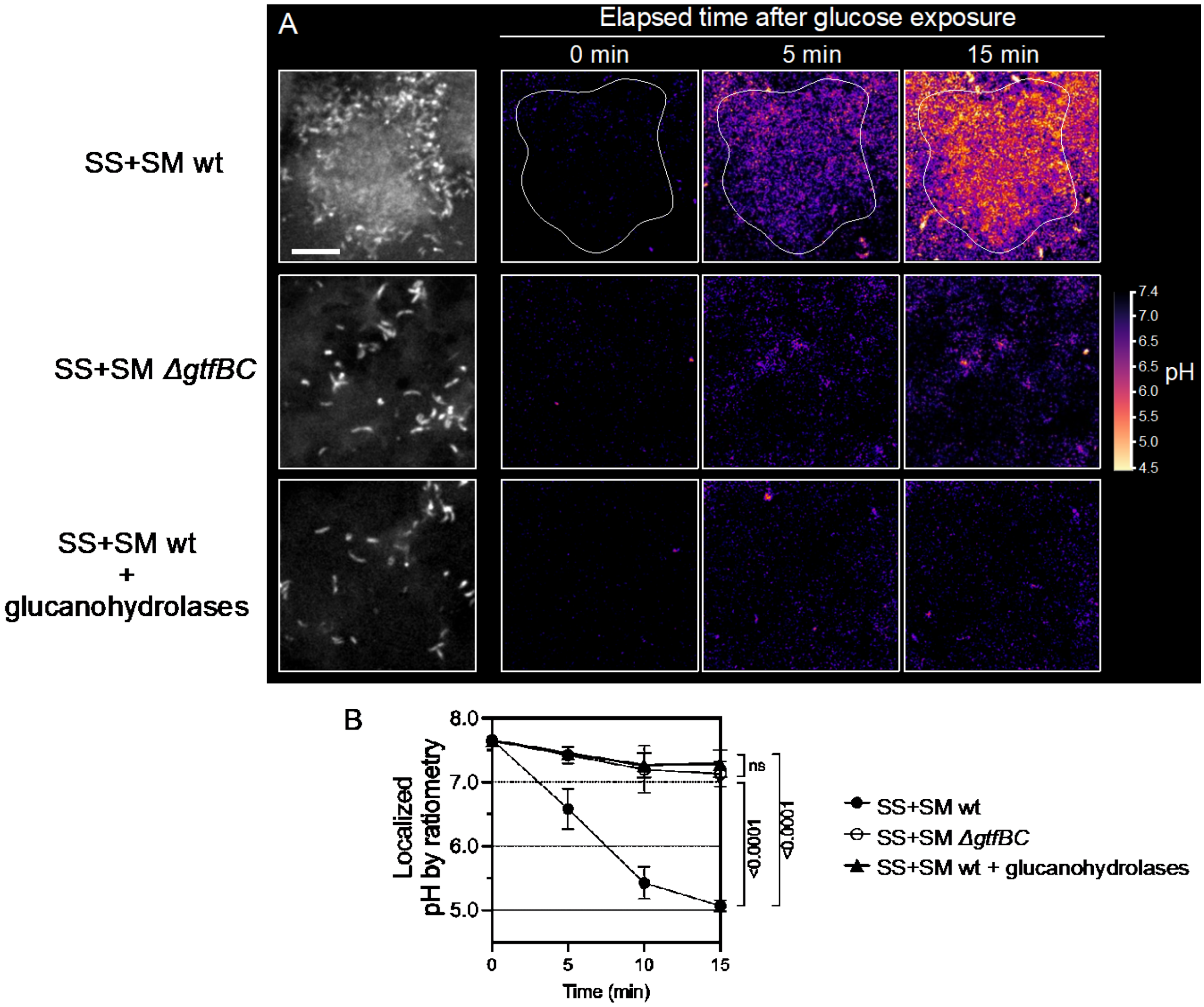

Supplementary Fig. 6 | *S. sputigena-S. mutans* biofilm causes dental caries (i.e., tooth decay) lesions on human enamel. (A) Multi-scale analyses of the human tooth-enamel underneath the experimental biofilms. Left, macroscopically demineralized, enamel caries lesions (brighter, chalky areas) developed on the enamel surface inflicted by *S. mutans* alone (SM), *S. mutans-P. salivae* co-culture (SM-PS), and *S. mutans-S. sputigena* co-culture (SM-SS); right, corresponding surface topography analysis showing microcavities formed on the enamel surface. The enamel surface topography is color-coded to visualize the microcavities. (B) Transverse microradiography of the human enamel surface underneath the biofilms. Average lesion depths of enamel demineralization caused by the biofilms are shown on the image. (C) Quantitative analysis of tooth surface topography (roughness), and tooth mineral analysis (mineral loss and lesion depth). Data are presented as mean ± standard deviation (n=4 biologically independent samples). Groups that do not share the same lowercase letter are significantly different from each other (p<0.05 by one-way analysis of variance with Tukey's multiple-comparison test). Roughness of enamel surface: SM vs. SM+SS, $p = 0.0029$; SM+PS vs. SM+SS, $p = 0.0123$. Mineral loss: SM vs. SM+SS, $p = 0.0015$; SM+PS vs. SM+SS, $p = 0.003$. Lesion depth: SM vs. SM+SS, $p = 0.0096$; SM+PS vs. SM+SS, $p = 0.005$.

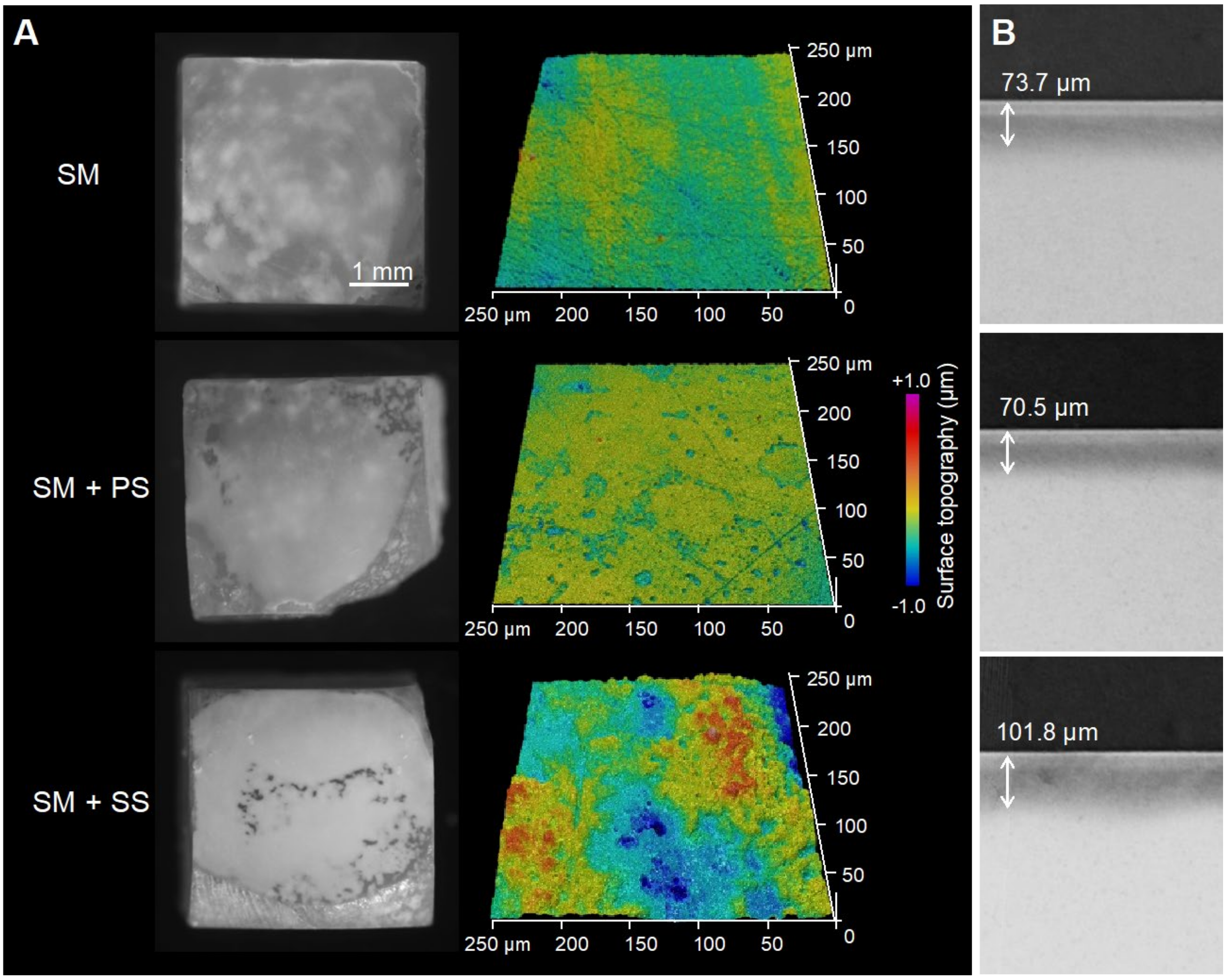

C

	Roughness of enamel surface (μm)	Mineral loss (% × μm)	Lesion depth (μm)
SM	0.09±0.03[a]	2680.0±553.0[a]	73.7±16.1[a]
SM+PS	0.18±0.14[a]	2335.0±507.7[a]	70.5±5.0[a]
SM+SS	0.51±0.16[b]	3757.5±145.7[b]	101.8±5.7[b]

Supplementary Fig. 7 | Flowchart of high-throughput sequencing data (MTG) pre-processing in the discovery ZOE 2.0 sample (n=300).

Eligible participants with clinical data	(n = **300**, # species = 6,914)	
↓	→	Exclusion of taxa:<br>viruses (# = 503)
Dataset including all non-viral taxa	(n = 300, # species = **6,411**, mean RPK per subject = 8,004,958)	
↓	→	Normalization:<br>RPK to TPM
Per-participant normalized dataset	(n = 300, # species = 6,411, mean TPM per subject = **8,000,000**)	
↓	→	Exclusion of taxa:<br>low prevalent (<10%) and<br>low abundant (<80 in TPM) species
Participant samples with informative species	(n = 300, # species = **418**, mean TPM per subject = **7,962,295**)	

Supplementary Table 1 | Bacterial species significantly associated with dental caries experience. The magnitude of association was measured by log-normal model coefficients (the increment of log-abundance or log-expression levels of each species per increment in the quantitative caries status), and statistical significance was determined by two-sided Wald tests and FDR-adjusted *p*-values. *p*, nominal *p*-values; *q*, FDR-adjusted *p*-values. Corresponding 95% confidence intervals are available in the source data file in the Supplementary Information. These 16 species were significantly associated with early childhood caries (ECC) experience defined as a quantitative localized trait (i.e., from the 5 tooth surfaces from which biofilm was collected) in shotgun metagenomics (DNA-seq.-MTG) analyses in the ZOE 2.0 study (n= # subjects =300). The identified species associations were replicated in metatranscriptomics (RNA-seq.-MTX) analyses, and for the person-level (i.e., entire mouth) equivalent quantitative trait of ECC in both metagenomics and metatranscriptomics analyses. Replication of these associations was further done in an independent sample of 116 similarly aged children. Underlined species were carried forward to *in vitro* assays and *in vivo* experiments.

	**Localized caries experience**								**person-level caries experience**							
	**MTG**				**MTX**				**MTG**				**MTX**			
	FDR-significant, discovery (n=300)	replication (n=116)			FDR-significant, discovery (n=297)	replication (n=116)			FDR-significant, discovery (n=300)	replication (n=116)			FDR-significant, discovery (n=297)	replication (n=116)		
bacterial species	log-normal coef. (*p*, *q*)	SD	Sig	FDR	log-normal coef. (*p*, *q*)	SD	Sig	FDR	log-normal coef. (*p*, *q*)	SD	Sig	FDR	log-normal coef. (*p*, *q*)	SD	Sig	FDR
<u>*Prevotella salivae*</u>	0.31 ($8.5x10^{-6}$, $4.9x10^{-4}$)	✓	✓	✓	0.32 ($1.3x10^{-5}$, $7.1x10^{-4}$)	✓	✓	✓	0.027 ($8.9x10^{-5}$, $2.5x10^{-3}$)	✓	✓	✓	0.032 ($5.3x10^{-6}$, $2.3x10^{-4}$)	✓	✓	✓
<u>*Streptococcus mutans*</u>	0.51 ($2.6x10^{-6}$, $2.2x10^{-4}$)	✓	✓	✓	0.34 ($1.0x10^{-3}$, $1.5x10^{-2}$)	✓	✓	✓	0.049 ($2.4x10^{-6}$, $2.5x10^{-4}$)	✓	✓		0.036 ($4.7x10^{-4}$, $8.0x10^{-3}$)	✓	✓	
<u>*Selenomonas sputigena*</u>	0.53 ($3.1x10^{-6}$, $2.2x10^{-4}$)	✓	✓	✓	0.51 ($1.1x10^{-4}$, $3.5x10^{-3}$)	✓	✓	✓	0.057 ($2.7x10^{-7}$, $7.8x10^{-5}$)	✓	✓		0.059 ($5.3x10^{-6}$, $2.3x10^{-4}$)	✓	✓	
Veillonella atypica	0.41 ($1.1x10^{-5}$, $4.9x10^{-4}$)	✓	✓	✓	0.29 (2.0x10-3, $2.2x10^{-2}$)	✓	✓	✓	0.040 ($1.0x10^{-5}$, $7.3x10^{-4}$)	✓	✓		0.029 (1.7x10-3, $1.9x10^{-2}$)	✓	✓	
Prevotella oulorum	0.51 ($1.5x10^{-7}$, $4.5x10^{-5}$)	✓	✓	✓	0.40 ($4.1x10^{-6}$, $3.1x10^{-4}$)	✓	✓	✓	0.045 ($2.3x10^{-6}$, $2.5x10^{-4}$)	✓	✓		0.041 ($2.2x10^{-6}$, $1.6x10^{-4}$)	✓		
Leptotrichia sp oral taxon 847	0.38 ($9.9x10^{-6}$, $4.9x10^{-4}$)	✓	✓	✓	0.33 ($2.2x10^{-6}$, $2.1x10^{-4}$)	✓	✓	✓	0.034 ($4.5x10^{-5}$, $2.1x10^{-3}$)	✓	✓		0.032 ($4.8x10^{-6}$, $2.3x10^{-4}$)	✓		
Lachnospiraceae bacterium oral taxon 082	0.30 ($1.1x10^{-4}$, $2.8x10^{-3}$)	✓	✓	✓	0.27 ($1.1x10^{-4}$, $3.5x10^{-3}$)	✓	✓	✓	0.026 ($3.8x10^{-4}$, $8.1x10^{-3}$)	✓			0.027 ($9.0x10^{-5}$, $3.1x10^{-3}$)	✓		
Leptotrichia sp oral taxon 223	0.29 ($6.3x10^{-5}$, $1.8x10^{-3}$)	✓	✓		0.30 ($1.3x10^{-5}$, $7.1x10^{-4}$)	✓	✓	✓	0.027 ($1.4x10^{-4}$, $3.6x10^{-3}$)	✓	✓		0.033 ($1.6x10^{-6}$, $1.6x10^{-4}$)	✓	✓	
<u>*Leptotrichia wadei*</u>	0.41 ($2.2x10^{-7}$, $4.5x10^{-5}$)	✓	✓		0.48 ($1.0x10^{-8}$, $4.0x10^{-6}$)	✓	✓	✓	0.039 ($3.7x10^{-7}$, $7.8x10^{-5}$)	✓			0.051 ($4.7x10^{-10}$, $1.8x10^{-7}$)	✓		
Leptotrichia sp oral taxon 498	0.57 ($9.1x10^{-7}$, $1.0x10^{-4}$)	✓	✓		0.56 ($6.6x10^{-7}$, $8.5x10^{-5}$)	✓	✓	✓	0.049 ($1.6x10^{-5}$, $9.4x10^{-4}$)	✓			0.052 ($2.5x10^{-6}$, $1.6x10^{-4}$)	✓		
Lachnoanaerobaculum saburreum	0.43 ($9.6x10^{-7}$, $1.0x10^{-4}$)	✓	✓		0.43 ($4.6x10^{-7}$, $8.5x10^{-5}$)	✓	✓	✓	0.039 ($5.0x10^{-6}$, $4.2x10^{-4}$)	✓			0.041 ($1.1x10^{-6}$, $1.6x10^{-4}$)	✓		
Prevotella sp oral taxon 306	0.37 ($4.3x10^{-5}$, $1.6x10^{-3}$)	✓	✓		0.34 ($6.1x10^{-4}$, $1.1x10^{-2}$)	✓	✓	✓	0.035 ($7.2x10^{-5}$, $2.2x10^{-3}$)	✓			0.037 ($1.3x10^{-4}$, $3.7x10^{-3}$)	✓		
Prevotella veroralis	0.37 ($6.4x10^{-5}$, $1.8x10^{-3}$)	✓	✓		0.37 ($2.7x10^{-4}$, $6.4x10^{-3}$)	✓	✓	✓	0.036 ($7.2x10^{-5}$, $2.2x10^{-3}$)	✓			0.037 ($1.7x10^{-4}$, $4.5x10^{-3}$)	✓		
Prevotella melaninogenica	0.40 ($4.8x10^{-5}$, $1.6x10^{-3}$)	✓	✓		0.32 ($5.3x10^{-4}$, $1.1x10^{-2}$)	✓	✓		0.037 ($7.4x10^{-5}$, $2.2x10^{-3}$)	✓			0.034 ($1.6x10^{-4}$, $4.3x10^{-3}$)	✓		
Stomatobaculum longum	0.36 ($1.6x10^{-3}$, $1.8x10^{-2}$)	✓	✓		0.36 ($1.1x10^{-3}$, $1.5x10^{-2}$)	✓			0.036 ($1.4x10^{-3}$, $1.7x10^{-2}$)	✓	✓		0.031 ($4.2x10^{-3}$, $3.9x10^{-2}$)	✓		
Centipeda periodontii	0.31 ($3.8x10^{-3}$, $3.1x10^{-2}$)	✓	✓		0.37 ($2.5x10^{-3}$, $2.6x10^{-2}$)	✓			0.030 ($3.9x10^{-3}$, $3.6x10^{-2}$)	✓			0.034 ($5.3x10^{-3}$, $4.7x10^{-2}$)	✓		

SD, same direction in discovery and replication samples; Sig, nominally statistically significant in the replication sample; FDR, multiple testing-corrected statistically significant in the replication sample

Supplementary Table 2 | Six pathways found to be associated with caries experience (localized, quantitative trait) after correction for multiple testing*. Four of these pathways involved one or more of the 16 significant species.

Pathway	pathway description/link	*p*	beta	pathway expression rank	% significant species	significant species in pathway
PWY-1042	glycolysis IV	2.9×10^{-4}	0.0558	3	23.2	*Cen. Periodontii, Lac. Saburreum, Lep. Wadei, Pre. Melaninogenica, Pre. Oulorum, Sel. Sputigena*
PWY-5100	pyruvate fermentation to acetate and lactate II	4.9×10^{-4}	-0.0394	9	7.7	*Lac. Saburreum*
UDPNAGSYN-PWY	UDP-N-acetyl-D-glucosamine biosynthesis I	5.5×10^{-4}	0.0308	42	9.8	*Cen. Periodontii, Lep. Wadei, Sel. Sputigena, Vei. Atypica*
PWY-6163	chorismate biosynthesis from 3-dehydroquinate	6.1×10^{-4}	0.0327	108	8.9	*Lac. Saburreum, Pre. Melaninogenica*
PWY-7234	inosine-5'-phosphate biosynthesis III	1.0×10^{-5}	-0.0813	83	0	none
PWY-6263	superpathway of menaquinol-8 biosynthesis II	1.4×10^{-4}	0.7359	266	0	none

* The magnitude of association was measured by log-normal model coefficients (the increment in the log-expression level of each pathway per increment in the quantitative caries status), and statistical significance was determined by two-sided Wald tests and FDR-adjusted *p*-values. *p*, nominal *p*-values; *q*, FDR-adjusted *p*-values. An FDR correction was implemented for testing 297 pathways. Corresponding 95% confidence intervals are available in the source data file in the Supplementary Information.

Supplementary Table 3 | Top species' genes significantly differentially expressed according to caries experience (FDR-adjusted *p*<0.05).

gene	species	*p*	direction	gene name
rnpB	*S. mutans*	2.4×10^{-6}	up	Rnase P RNA component class B
glnA	*S. mutans*	3.0×10^{-3}	up	type I glutamate–ammonia ligase
F5989_RS09410	*S. mutans*	3.0×10^{-3}	up	glucose-1-phosphate adenylyltransferase
gtfC	*S. mutans*	3.6×10^{-3}	up	glucosyltransferase GtfC
F5989_RS03555	*S. mutans*	6.0×10^{-3}	up	GBS Bsp-like repeat-containing protein
F5989_RS06655	*S. mutans*	8.2×10^{-3}	up	NAD-dependent succinate-semialdehyde dehydrogenase
lpdA	*S. mutans*	8.5×10^{-3}	up	dihydrolipoyl dehydrogenase
F5989_RS01985	*S. mutans*	8.8×10^{-3}	up	ATP-dependent Clp protease ATP-binding subunit
F5989_RS08255	*S. mutans*	9.0×10^{-3}	down	23S ribosomal RNA
rnpB	*P. salivae*	1.1×10^{-3}	up	Rnase P RNA component class A
HMPREF9145_RS11155	*P. salivae*	4.0×10^{-3}	down	TolC family protein
HMPREF9145_RS11160	*P. salivae*	4.1×10^{-3}	down	efflux RND transporter permease subunit
pckA	*P. salivae*	6.1×10^{-3}	up	phosphoenolpyruvate carboxykinase (ATP)
HMPREF9145_RS09270	*P. salivae*	7.0×10^{-3}	up	OmpA family protein
tuf	*P. salivae*	1.8×10^{-2}	up	elongation factor Tu
rpsM	*L. wadei*	7.3×10^{-5}	up	30S ribosomal protein S13

No significantly differentially expressed gene in dental caries was found for *Selenomonas sputigena* after correction for multiple comparisons. The magnitude of association was measured by log-normal model coefficients (the increment in the log-expression level of each gene-species combination per increment in quantitative caries status), and statistical significance was determined by two-sided Wald tests and FDR-adjusted p-values. *p*, nominal *p*-values. Corresponding 95% confidence intervals are available in the source data file in the Supplementary Information.

**Supplementary Table 4** | Significant (FDR *p*<0.05) gene-gene expression interactions between *S mutans* and *S sputigena.*					
S mutans gene	*S sputigena* gene	*p*	direction	*S mutans* gene name	*S sputigena* gene name
F5989_RS09410	SELSP_RS02150	$1.6x10^{-7}$	negative	glucose-1-phosphate adenylyltransferase	YDG domain-containing protein
F5989_RS09410	eno	$2.0x10^{-6}$	negative	glucose-1-phosphate adenylyltransferase	phosphopyruvate hydratase
F5989_RS09410	gap	$3.0x10^{-6}$	negative	glucose-1-phosphate adenylyltransferase	type I glyceraldehyde-3-phosphate dehydrogenase
F5989_RS09410	SELSP_RS01640	$3.7x10^{-6}$	negative	glucose-1-phosphate adenylyltransferase	flagellin
glgD	rnpB	$1.9x10^{-5}$	positive	glucose-1-phosphate adenylyltransferase subunit GlgD	Rnase P RNA component class A
F5989_RS09410	SELSP_RS01225	$2.2x10^{-5}$	negative	glucose-1-phosphate adenylyltransferase	iron transporter
F5989_RS09410	SELSP_RS11020	$2.3x10^{-5}$	negative	glucose-1-phosphate adenylyltransferase	DUF3793 family protein
F5989_RS09410	SELSP_RS08375	$4.1x10^{-5}$	negative	glucose-1-phosphate adenylyltransferase	malate dehydrogenase
F5989_RS09410	SELSP_RS08335	$7.2x10^{-5}$	negative	glucose-1-phosphate adenylyltransferase	S-layer homology domain-containing protein
hprK	ssrS	$1.1x10^{-4}$	positive	HPr(Ser) kinase/phosphatase	6S RNA
F5989_RS09410	SELSP_RS04605	$1.4x10^{-4}$	negative	glucose-1-phosphate adenylyltransferase	acyl-CoA mutase large subunit family protein
F5989_RS09410	SELSP_RS01200	$1.5x10^{-4}$	negative	glucose-1-phosphate adenylyltransferase	DUF4198 domain-containing protein
glgD	SELSP_RS02150	$3.5x10^{-4}$	negative	glucose-1-phosphate adenylyltransferase subunit GlgD	YDG domain-containing protein

The magnitude of association was measured by log-normal model coefficients (the increment in the log-expression level of each gene-species combination interaction per increment in the quantitative caries status), and statistical significance was determined by two-sided Wald tests and its FDR-adjusted p-values. *p*, nominal *p*-values. Corresponding 95% confidence intervals are available in the source data file in the Supplementary Information.

Supplementary Table 5 | Descriptive information of the discovery (n = 300 children) and replication (n = 116 children) samples used in human microbiome taxonomic discovery. Caries experience was defined using sensitive caries lesion detection criteria (i.e., including early-stage lesions, ICDAS≥1) among the surfaces from which plaque biofilm was collected (i.e., localized experience) and the entire dentition (i.e., person-level disease experience).

	**n**		**age**	**sex**		**race/ethnicity**			**caries experience**	
	MTG	MTX	months (SD)	male, n (%)	female, n (%)	Non-Hispanic white	African American	Hispanic/ other	localized, dmfs (SD)	person-level, dmfs (SD)
discovery	300	297	52 (8)	155 (52)	145 (48)	89 (30%)	113 (38%)	98 (33%)	0.9 (1.5)	14 (16)
replication	116	116	55 (8)	53 (46)	63 (54)	18 (16%)	52 (45%)	46 (40%)	0.7 (1.2)	8.9 (15)

ICDAS, the international caries detection and classification system wherein 1 is first visual change in enamel ("initial lesion"); MTG, metagenomics, whole genome shotgun sequencing data; MTX, metatranscriptomics, RNA-seq data; dmfs, the number of decayed, missing and filled (restored) tooth surfaces due to dental caries. Age, sex, and caries experience information is presented for participants with MTG data.
