# Supplementary material for: Selenomonas sputigena acts as a pathobiont mediating spatial structure and biofilm virulence in early childhood caries
Source: Nat Commun. 2023 May 22;14:2919. doi: 10.1038/s41467-023-38346-3 (PMC10202936; doi:10.1038/s41467-023-38346-3)
Supplement: Supplementary file 3 — Description of Additional Supplementary Files [file 41467_2023_38346_MOESM3_ESM.docx]

**File Name**: Supplementary Movie 1

**Description**: Surface motility of S. sputigena cells on saliva-coated hydroxyapatite disc (sHA) surface is characterized by a tumbling, multi-directional motion while bacteria remain attached to the sHA surface.

**File Name**: Supplementary Movie 2

**Description**: Surface motility of S. sputigena cells on sHA when co-cultured with S. mutans, assessed with real-time live imaging and quantified using computational motion tracking. Most S. sputigena cells adjacent to S. mutans did not move or only moved for short distances, whereas those with no S. mutans nearby moved actively and for longer distances.

**File Name**: Supplementary Movie 3

**Description**: Three-dimensional reconstruction of confocal images and quantitative analysis of the spatial distribution of bacterial cells. The reconstruction reveals a distinctive multicellular honeycomb-like superstructure formed by S. sputigena guided by matrix scaffolding.
