# Supplementary material for: Selenomonas sputigena acts as a pathobiont mediating spatial structure and biofilm virulence in early childhood caries
Source: Nat Commun. 2023 May 22;14:2919. doi: 10.1038/s41467-023-38346-3 (PMC10202936; doi:10.1038/s41467-023-38346-3)
Supplement: Supplementary file 7 — Reporting summary [file 41467_2023_38346_MOESM7_ESM.pdf]

nature portfolio

Corresponding author(s): Kimon Divaris, Di Wu, Hyun Koo

Last updated by author(s): Apr 7, 2023

Reporting Summary

Nature Portfolio wishes to improve the reproducibility of the work that we publish. This form provides structure for consistency and transparency in reporting. For further information on Nature Portfolio policies, see our Editorial Policies and the Editorial Policy Checklist.

Statistics

For all statistical analyses, confirm that the following items are present in the figure legend, table legend, main text, or Methods section.

n/a	Confirmed	
☐	☒	The exact sample size (*n*) for each experimental group/condition, given as a discrete number and unit of measurement
☐	☒	A statement on whether measurements were taken from distinct samples or whether the same sample was measured repeatedly
☐	☒	The statistical test(s) used AND whether they are one- or two-sided<br>*Only common tests should be described solely by name; describe more complex techniques in the Methods section.*
☐	☒	A description of all covariates tested
☐	☒	A description of any assumptions or corrections, such as tests of normality and adjustment for multiple comparisons
☐	☒	A full description of the statistical parameters including central tendency (e.g. means) or other basic estimates (e.g. regression coefficient) AND variation (e.g. standard deviation) or associated estimates of uncertainty (e.g. confidence intervals)
☐	☒	For null hypothesis testing, the test statistic (e.g. *F*, *t*, *r*) with confidence intervals, effect sizes, degrees of freedom and *P* value noted<br>*Give P values as exact values whenever suitable.*
☒	☐	For Bayesian analysis, information on the choice of priors and Markov chain Monte Carlo settings
☒	☐	For hierarchical and complex designs, identification of the appropriate level for tests and full reporting of outcomes
☐	☒	Estimates of effect sizes (e.g. Cohen's *d*, Pearson's *r*), indicating how they were calculated

Our web collection on statistics for biologists contains articles on many of the points above.

Software and code

Policy information about availability of computer code

Data collection	No software was used for data collection. See data analysis section for information of all software used for data analysis.
Data analysis	Illumina sequencing data were demultiplexed using Ilumina Bcl2Fastq 2.20.0 (Illumina, Inc. San Diego, CA, USA.). Quality control of the demultiplexed sequencing reads was verified by FastQC (Babraham Institute. Cambridge, UK). Adapters were trimmed using Trim Galore (Babraham Institute. Cambridge, UK). Paired-end reads were joined with vsearch 1.10.2. Estimates of taxonomic composition, gene family, path abundance, and path coverage were produced from the remaining reads using Kracken2/Bracken and a custom database including human, fungal, bacterial, and the expanded Human Oral Microbiome Database (eHOMD) genomes. For targeted MTX analyses, reads reads were aligned to the relevant reference transcriptome of the top 4 species using STAR (v.2.7.10b) and subsequently quantified via Salmon. (v1.10.1) Metabolic activity was measured using calView™ software (Version 1.0.33.0, © 2015 SymCel, Sverige AB). Fluorescent signals were substracted from each other for biofilm imaging using applied channel subtraction using the Image Calculator in ImageJ Fiji version 2.11.0 (https://imagej.net/Fiji). Computational image processing and quantitative analysis were performed using BiofilmQ software version 0.2.2 (https://drescherlab.org/data/biofilmQ). To track single-cell bacterial motility in real time, we performed computational single-particle tracking and generated time-resolved trajectories using the TrackMate plugin in ImageJ Fiji version 2.11.0 (https://imagej.net/software/fiji/). Biofilm visualization was performed using maximum intensity projection and 3D surface rendering in ImageJ Fiji. Confocal microscopy images of enamel surface topography and roughness were obtained using ConfoMap software version 7.3.7984 (https://asset-downloads.zeiss.com/catalogs/download/mic/fb736fea-6b1b-4a57-a1a0-1bc4861286f5/EN_product-info_ConfoMap_rel-1.0.pdf). Microradiography images obtained with the MTR-D system were obtained using TMR software version 3.0.0.18 (https://www.inspektor.nl/irsresearch.html#). R code was used for all other statistical analyses of microbiome features. The code has been made available at the following location: DOI: 10.5281/zenodo.7707297

For manuscripts utilizing custom algorithms or software that are central to the research but not yet described in published literature, software must be made available to editors and reviewers. We strongly encourage code deposition in a community repository (e.g. GitHub). See the Nature Portfolio guidelines for submitting code & software for further information.

Data

Policy information about availability of data

All manuscripts must include a data availability statement. This statement should provide the following information, where applicable:

- Accession codes, unique identifiers, or web links for publicly available datasets
- A description of any restrictions on data availability
- For clinical datasets or third party data, please ensure that the statement adheres to our policy

Raw DNA and RNA sequence data for ZOE 2.0 have been deposited and are publicly available as part of dbGaP accession phs002232.v1.p1 "TOPDECC-Trans-omics for Precision Dentistry and Early Childhood Caries: Genome-Wide Genotyping (CIDR) and Microbiome in the ZOE 2.0 Study" and the Sequence Read Archive (SRA) as BioProject 671299 (PRJNA671299; dbGaP: phs002232; https://www.ncbi.nlm.nih.gov/bioproject/671299). Raw DNA and RNA sequence data for ZOE pilot (the replication sample) have been deposited and are publicly available as part of BioProject 843091 "ZOE 2.0 pilot study" (PRJNA843091; https://www.ncbi.nlm.nih.gov/bioproject/PRJNA843091). Microbiome taxonomy (MTG and MTX) and pathway information for the entire biofilm microbial community, as well as targeted MTX data for the four top species used in this study have been deposited and are publicly available alongside metadata (i.e., demographic and clinical phenotype information) via the Carolina Digital Repository and accession number 5d86p890x (https://cdr.lib.unc.edu/concern/data_sets/5d86p890x). Reference genomes were obtained from the expanded Human Oral Microbiome Database (https://www.homd.org/). The source data file used to generate (extended) the figures and the tables is available in the Supplementary Information.

Field-specific reporting

Please select the one below that is the best fit for your research. If you are not sure, read the appropriate sections before making your selection.

- [x] Life sciences
- [ ] Behavioural & social sciences
- [ ] Ecological, evolutionary & environmental sciences

For a reference copy of the document with all sections, see nature.com/documents/nr-reporting-summary-flat.pdf

Life sciences study design

All studies must disclose on these points even when the disclosure is negative.

Sample size	The sample size for taxonomic discovery was determined based on a convenience sample representing ~5% of the parent cohort study (n=300 out of ~6,400 participants) and was selected as a case-control sample of caries cases:control among the first enrolled/examined participants. This sample was multiple times larger than previous studies reporting taxonomic discovery employing WGS shotgun and/or RNA-seq. in dental caries, e.g., PMID: 33239396 included 47 participants, PMID: 30671194 included 30 participants, and PMID: 32423437 included 20 participants. The replication sample of 116 participants was fixed and was not based on based on a calculation but rather availability of funds to generate MTG and MTX data to determine feasibility and fidelity of the experimental approach.<br>For the in vivo study, sample size was calculated as follows: σ (20%) and Δ (25%) were estimated from preliminary data and our previously published work using similar co-infection regimen. The sample size (8 animals per group) provides 80% power to detect differences between groups at an alpha level of 0.05. For in vitro studies, sample sizes were determined based on established lab protocols and previous publications (e.g., PMID: 32170131, 30065293, 25501477) using the same method to ensure feasibility and reproducibility.
Data exclusions	MTX data were not available for 3 participants in the ZOE 2.0 study (discovery sample) leaving an analytical sample of 297 for that analysis; meanwhile, MTG and MTX data were not available for 2 ZOE pilot (replication sample) participants, leaving analytical samples of 116 for these analyses.
Replication	Replication of the identified associations of taxonomic abundance in supragingival biofilm microbiome data were sought across 8 different analyses. These involved MTG and MTX data, localized and person-level caries experience traits, as well as a discovery and a replication sample. As evidence of replication, we considered, in order of ascending importance, 1) directional consistency of the estimate of association, 2) nominal significance, or 3) FDR-level significance in the replication sample. Species that were FDR-significant in all 4 models in the discovery sample and were at least nominally significant for localized disease experience in MTG data were termed "significant species". This set of species with high-confidence evidence of association from multiple traits, MTG and MTX data, and from all 416 study participants were prioritized for reporting and were candidates for consideration in the experimental validation pipeline. Elsewhere, three independent experiments were performed unless otherwise stated. All attempts at replication were successful.
Randomization	There were no human experimental group or intervention allocation in this study. Randomization of animals was undertaken at the experimental aspect of the study.
Blinding	Blinding is not relevant to this observational study because all participants underwent clinical dental examinations prior to the conduct of microbiome analyses and the latter could not have possibly influenced clinical examination findings. For the animal experimental study, investigators were masked to experimental group (i.e., infection) allocations during the infection, sampling, and assessment stages, by using color-coded samples.

Reporting for specific materials, systems and methods

We require information from authors about some types of materials, experimental systems and methods used in many studies. Here, indicate whether each material, system or method listed is relevant to your study. If you are not sure if a list item applies to your research, read the appropriate section before selecting a response.

Materials & experimental systems

n/a	Involved in the study	
x		Antibodies
x		Eukaryotic cell lines
x		Palaeontology and archaeology
	x	Animals and other organisms
	x	Human research participants
	x	Clinical data
x		Dual use research of concern

Methods

n/a	Involved in the study	
x		ChIP-seq
x		Flow cytometry
x		MRI-based neuroimaging

Animals and other organisms

Policy information about studies involving animals; ARRIVE guidelines recommended for reporting animal research

Laboratory animals	Laboratory animals were 15 day-old female Sprague-Dawley rat pups, specific-pathogen-free grade, that were purchased with dams (8 pups per dam) from Harlan Laboratories (Indianapolis, IN, USA)
Wild animals	There were no wild animals involved in this study.
Field-collected samples	No field samples were collected as part of the study.
Ethics oversight	The in vivo experimental study was reviewed and approved by the Institutional Animal Care and Use Committee of the University of Pennsylvania (IACUC#805735).

Note that full information on the approval of the study protocol must also be provided in the manuscript.

Human research participants

Policy information about studies involving human research participants

Population characteristics	Participants in the discovery cohort (ZOE 2.0 study) were 300 children with mean age 4.5 yrs (52 months), 48% female, and of mixed race/ethnicity (i.e., 38% African-American, 33% Hispanics, and 30% non-Hispanic whites), selected as a 5% subset of the parent cohort of the ZOE 2.0 study, 1:1 case-control ratio for established person-level dental caries experience. Participants were sequenced in two batches. Two initially selected participants who did not produce microbiome sequencing or clinical data in the first batched were replaced in the second batch, to maintained a discovery sample size of 300. The replication sample comprised 116 preschool-age children from the same population (i.e., public preschools in North Carolina), who were of similar age (55 months), 54% were female, and also of mixed race/ethnicity (i.e., 45% African American, 40% Hispanics, and 16% non-Hispanic whites). Two participants out of an intial sample of 118 were excluded due to insufficient reads produced in MTG/MTX. The distribution of male/female participants was balanced in the study, with a total of 208 children (in both ZOE 2.0 and ZOE pilot) in each stratum. This information was obtained from questionnaires completed by parents or legals guardians which was then cross-checked with biological sex data available via genotyping.
Recruitment	Participants of both ZOE 2.0 (discovery sample) and ZOE pilot (replication sample) were a community-based sample of children attending public preschools (i.e., Head Start programs/centers) in North Carolina. Children were eligible for the study based on their attendance in public preschools in North Carolina during the study period 2016-2019 in 80 out of 100 counties of the state. All children in this state-wide sample of 3-5-year-olds attending public preschools were eligible for study participation, as long as they had a caregiver at least 18 years of age who understood the study documents and agreed to participate. No exclusion criteria were applied, as long as the aforementioned inclusion criteria were met. Recruitment took place via distribution of study materials to all families via preschool children staff at all participating centers--families then received and completed study material which was then collected by study staff prior to the study visit at their children's school. Given the all-inclusive recruitment and sampling strategy of the parent study, we believe that there are no appreciable systematic errors biasing this study's findings. Among approximately 13,000 invitation packets distributed, a little over 8,000 were received and were completed, and 6,400 of participants participated in all study procedures including clinical examinations and biospecimen collection. Families were provided with a $20 gift card as compensation for their time. The most common reason for not completing the study visits was being absent from school the day that the study team went to that specific school to conduct research activities. A flowchart of eligibility and enrollment of the parent study is presented as Figure in the cohort profile publication with PMID: 33139633. The 5% subset of ZOE 2.0 children who were selected to have biofilm analyses carried out were among the first 300 (150 with dental caries and 150 without dental caries) who were examined, as part of a feasiblity sub-study. Children in the replication sample were a convenience sample of children from the same population that contributed to studies supporting feasibility and fidelity of procedures and protocols developed for and employed in the parent study.
Ethics oversight	Human observational data and analyses received approval (#14-1992) from the University of North Carolina-Chapel Hill Office of Human Research Ethics Institutional Review Board on September 18, 2014. Legal guardians of all children provided written informed consent for participation in the study. All research was performed in accordance with the Declaration of Helsinki.

Note that full information on the approval of the study protocol must also be provided in the manuscript.

Clinical data

Policy information about clinical studies

All manuscripts should comply with the ICMJE guidelines for publication of clinical research and a completed CONSORT checklist must be included with all submissions.

Clinical trial registration	This was an observational study and thus was not registered as a clinical trial.
Study protocol	Clinical data collection protocol reported in Ginnis et al. 2019, PMID: 30838597; biofilm collection protocol reported in Divaris et al., 2019, PMID: 30838598.
Data collection	Data collection for the discovery sample (ZOE 2.0 study) took place between 2016-2019 across the state of North Carolina and in participating public preschool centers (i.e., Head Start). The cohort profile manuscript including details of data collection is reported in Divaris et al. 2020 PMID: 33139633.
Outcomes	Clinical endpoints measured in the ZOE studies were dental caries experience using modified ICDAS criteria for caries lesion detection. Recording of clinical measures of caries experience was done by 10 trained and calibrated clinical examiners at participating children's public preschool classroom. Caries experience in the ZOE 2.0 and the ZOE pilot studies was defined both at the local level (i.e., from the 5 surfaces where dental plaque biofilm was harvested from) and the person-level (i.e., considering all 88 tooth surfaces of a child's dentition). Details of the clinical endpoint definitions are provided in the study's clinical protocol Ginnis et al. 2019 PMID: 30838597 and a description of these endpoints in the entire cohort is provided in the cohort profile by Divaris et al. 2020 PMID: 33139633.
