# Supplementary material for: Selenomonas sputigena acts as a pathobiont mediating spatial structure and biofilm virulence in early childhood caries
Source: Nat Commun. 2023 May 22;14:2919. doi: 10.1038/s41467-023-38346-3 (PMC10202936; doi:10.1038/s41467-023-38346-3)
Supplement: Supplementary file 8 — Source Data [file 41467_2023_38346_MOESM8_ESM.zip › Supplementary_information_raw_data_legends.rtf]

Fig2_and_EFig1Zoe2, discovery data set; zoe1, validation data set; coef, log-normal model coefficients of the corresponding phenotype; se, the standard error of coef; lb, 95% confidence lower bound;  ub, 95% confidence upper bound; p, nominal p-value; q, FDR-adjusted p-valueFig3_DNA_health, Fig3_DNA_disease, Fig3_RNA_health, Fig3_RNA_diseaseCorrelation coefficients of each species pair for each caries status and the DNA/RNA type. Each row represents the same set of species in the column head in the order.EFig2Coef.loc, log-normal model coefficients of the localized caries experience; p, the corresponding nominal p-value; q, the corresponding FDR-adjusted p-value; star, q < 0.05; ETable1mD, log-normal model with localized caries experience as the main exposure and species abundance (DNA) as the outcome; mR, same as mD but with the species expression level (RNA) as the outcome;tD and tR; same model with mD and mR but with the person-level caries experience as the main exposure; .c, the coefficient and the confidence interval (nominal p-value, FDR-adjusted p-value); .val = FDR, FDR < 0.05; .val=sig, p < 0.05; .val=SD, coefficients have the same direction across the four models (mD, mR, tD, and tR); FDR (sig, SD), the number of FDR < 0.05 (p<0.05, SD) among the four models; p, the p-value in mD.c.ETable2beta, the log-normal model coefficient; p, nominal p-value of beta; se, standard error of beta; ci_lb (ci_ub), the lower (upper) bound of the 95% confidence interval of beta; expression rank, the rank of each pathway in terms of the expression level among all pathways; % sig species, for each pathway the percentage of expression level contributed by the significant species; sig species, the significant species that contributes to each pathway expression.ETable3Coef, the main effect coefficient of the linear model (the expression level of each gene-species combination as the main exposure and the binary disease status as the outcome); se, the standard error of coef; ci_lb (ci_ub), the 95% confidence interval lower bound (upper bound) of coef; p, the nominal p-value of testing the coefficients being zero; direction, + for coef > 0.ETable4.int (or .interaction), the interaction term; .Ss, the main effect term for S. Sputigena;  .Sm, the main effect term for S. mutans; Coef, the interaction effect coefficient of the linear model (the expression levels of two gene-species combinations and their interactions as the predictors and the binary disease status as the outcome); se, the standard error of coef; ci_lb (ci_ub), the 95% confidence interval lower bound (upper bound) of coef; p, the nominal p-value of testing the coefficients being zero; 
